# One-step upgrading of bio-based furfural to γ-valerolactone *via* HfCl_4_-mediated bifunctional catalysis†

**DOI:** 10.1039/d1ra05637a

**Published:** 2021-11-02

**Authors:** Mingrui Li, Yixuan Liu, Xialing Lin, Jinyu Tan, Song Yang, Hu Li

**Affiliations:** State Key Laboratory Breeding Base of Green Pesticide & Agricultural Bioengineering, Key Laboratory of Green Pesticide & Agricultural Bioengineering, Ministry of Education, State-Local Joint Laboratory for Comprehensive Utilization of Biomass, Center for R&D of Fine Chemicals, Guizhou University Guiyang Guizhou 550025 China hli13@gzu.edu.cn jhzx.msm@gmail.com

## Abstract

γ-Valerolactone (GVL) is an attractive biomass-derived platform molecule that plays an important role in the production of biofuels and biopolymers. The synthesis of GVL from renewable biomass and its derivatives has great application prospects but also presents challenges due to the multiple conversion steps involved. Here, a HfCl_4_-mediated acid–base bifunctional catalytic system was developed, which was demonstrated to be efficient for upgrading furfural (FF) to GVL in a single pot with unprecedented performance. The Lewis acidity of Hf^4+^ and moderate basicity of HfO(OH)_2_·*x*H_2_O, and strong Brønsted acidity of HCl *in situ* generated from HfCl_4_ hydrolysis were found to play a synergistic role in the cascade reaction processes, mainly contributing to the pronounced catalytic activity. The effects of the key reaction parameters, such as the catalyst dosage, reaction time, and temperature, on GVL production were optimized by response surface methodology. It is worth mentioning that the recovered catalyst after thermal treatment could be directly used for the hydrogen transfer processes, like FF-to-furfuryl alcohol conversion. This catalytic strategy opens a new avenue for the selective conversion of biomass feedstocks involving multiple steps and complex processes.

## Abstract 2

The one-pot multi-step conversion of biomass-derived furfural to γ-valerolactone (GVL) with 64.5% yield is achieved *via* acid–base bifunctional catalysis enabled by HfCl_4_ in 2-propanol, and the recovered solid is active for transfer hydrogenation.

## Graphical abstract

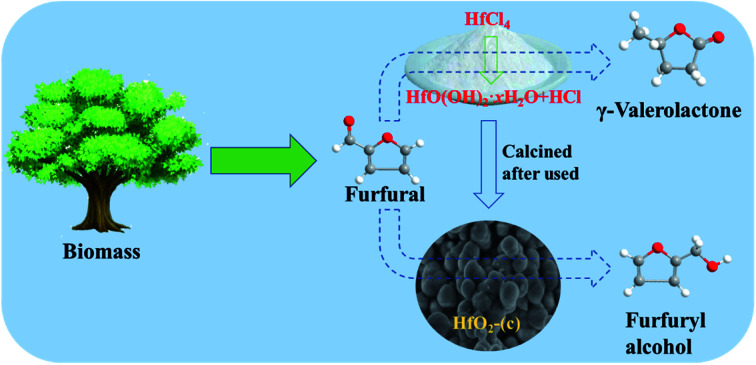

## 1. Introduction

**Scheme 1:**
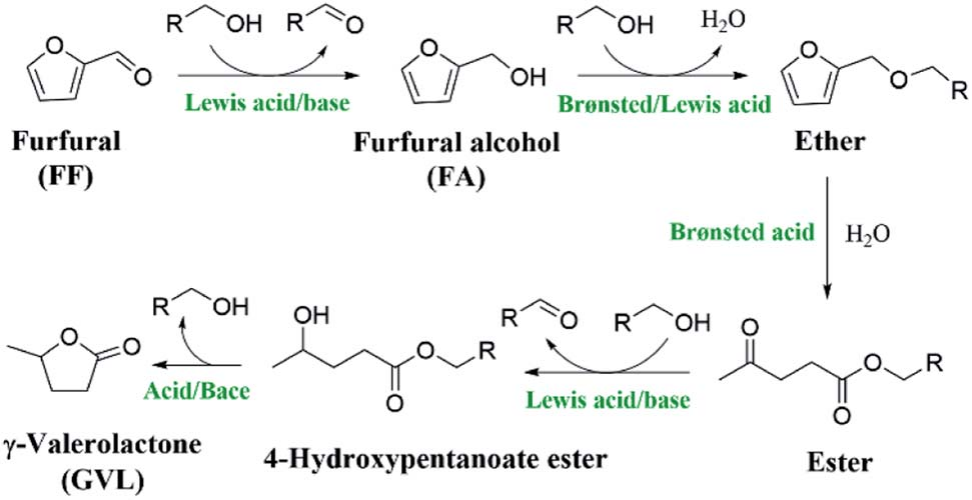
Process of synthesizing GVL from FF (R = alkyl group).

With the increasing demand for fine chemicals and fuels in modern society, the excessive exploitation and consumption of nonrenewable fossil energy resources are getting worse. There is thus an urgent need to seek renewable energy substitutes.^1,2^ Biomass is the most abundant organic carbon source and is widely distributed, which make it often used to produce biofuels and various valuable chemicals.^3–6^ Among the biomass feedstocks, C5 sugars (*e.g.*, arabinose and xylose) obtained from hemicellulose can be converted into furfural (FF), furfuryl alcohol (FA), and levulinic acid, which can be further upgraded to γ-valerolactone (GVL), an important and valuable chemical.^7–9^ GVL is considered a green solvent, biofuel additive, and biopolymer precursor because of its high lightning and low melting point.^10–14^ The process of preparing GVL by the hydrogenation of levulinic acid usually requires dangerous high-pressure H_2_ and the use of expensive noble metals as catalysts,^15–19^ which is not in line with the concept of energy-saving green chemistry. FF as an important biomass derivative has been reported to be able to successfully synthesize GVL in one pot.^20^ The synthesis of GVL from FF usually uses formic acid or alcohol as a hydrogen source instead of high-pressure hydrogen, which makes the process safer and greener. Moreover, by completing the process through a one-pot method, it is possible to effectively reduce the chemical waste caused by each step of the separation and purification, which makes it a more efficient and energy-saving process. Despite this, there are only limited reports on the synthesis of GVL from FF in one pot.^21–23^ The main barrier could be that the selective transformation of FF into GVL requires multiple steps (Scheme 1), including hydrogenation, hydrolysis ring-opening, partial hydrogenation, cyclization, and other steps (*e.g.*, etherification, esterification, and lactonization), in which a specific active site is needed for each step of the reaction.^9,24,25^ In addition, the reaction process is often accompanied by the formation of humin,^26–28^ making it far from ideal for commercialization.

In line with this, many bifunctional catalysts (*e.g.*, Au/ZrO_2_ + ASM-5, Hf-MOF-808 + Al-beta, Zr-HY + Al-HY, CuAl + H-ZSM-5, HZ-ZrP, DUT-67(Hf), HPW/Zr-beta, and ZrO_2_-SBA-15) have been developed to catalyze the conversion of FF to GVL.^20–23,29–35^ The selective conversion of FF to GVL requires two key catalytic transfer hydrogenation (CTH) reactions, which can be achieved by Lewis acid–base sites.^36–41^ In particular, the strength of Lewis acid–base sites was found to affect the yield of GVL. Lewis acid–base sites of a medium strength are more conducive to the occurrence of CTH reactions.^33^ In addition, the Brønsted acid site is also essential for the ring-opening reaction.^31^ In the reported studies, the non-noble metals that catalyze the cascade transformation of FF into GVL are mostly Zr-based catalysts,^26–28^ while there are few reports on Hf-based catalysts.^30^ Although these prepared catalysts show good catalytic activity for the conversion of FF to GVL, they have some disadvantages too that cannot be ignored. For instance, the preparation of the catalyst requires many complicated steps, while some may not require many steps but a long preparation cycle or time is necessary.^32,34^ Therefore, it is still desirable to develop low-cost, high-efficiency, and easily available catalysts for the conversion of FF to GVL.

In this work, a HfCl_4_-mediated acid–base bifunctional catalytic system was developed, which could effectively upgrade FF to GVL in one pot. The yield of GVL could reach 64.2% at 453 K in 8 h. It was found that the Lewis acid (HfCl_4_) and the *in situ*-formed Lewis base species HfO(OH)_2_·*x*H_2_O in medium strength were conducive to promoting FF and isopropyl levulinate (IPL) to undergo the CTH process, while the strong Brønsted acid (HCl) generated by HfCl_4_ hydrolysis made the furan ring open easily. Overall, the acid–base sites in the bifunctional catalyst were found to play a synergistic role in the efficient conversion of FF to GVL. The effects of the reaction temperature, reaction time, hydrogen donor, and catalyst dosage on the catalyst performance were studied, and further optimized by response surface methodology (RSM). In addition, the reaction kinetics and involved reaction mechanisms were also studied. Interestingly, the recovered catalyst calcined at 723 K for 6 h could effectively catalyze the transfer hydrogenation of FF to FA.

## 2. Experimental

### 2.1 Materials

Furfural (C_5_H_4_O_2_, >99%), naphthalene (C_10_H_8_, >99%), furfuryl alcohol (C_5_H_6_O_2_, >99%), γ-valerolactone (C_5_H_8_O_2_, >98%), methanol (CH_4_O, 99%), ethanol (C_2_H_6_O, 99%), 2-propanol (C_3_H_8_O, 99%), 2-butanol (C_4_H_10_O, 99%), 2-pentanol (C_5_H_12_O, 99%), ethyl acetate (C_4_H_8_O_2_, 99%), hafnium chloride (HfCl_4_, 99%), and ethyl levulinate (C_7_H_12_O_3_, 98%) were procured from Shanghai Aladdin Industrial Inc. All other reagents were of analytical grade and used without further treatment.

### 2.2 Catalytic conversion of FF to GVL

The conversion of FF to GVL was conducted in a 15 mL stainless steel reactor. Typically, 1 mmol FF (0.096 g), 15 mg naphthalene (internal standard), 0.015–0.06 mol% HfCl_4_ (1 g corresponding to 0.3 mol% HfCl_4_), and 6 mL 2-propanol were added to the reaction kettle. Then, the kettle was sealed, followed by transferring into a pre-heated oil bath to the desired temperature, and reacting for a certain time under magnetic stirring. After the reaction was finished, the reaction kettle was cooled to room temperature in a short time, and the mixture was filtered by a filter membrane, which was then analyzed by gas chromatography (GC) and gas chromatography-mass spectrometry (GC-MS).

### 2.3 Analytical method

The substances contained in the resulting liquid mixture were identified by a GC-MS system (Agilent 6890-5973) equipped with a 5973 MS mass spectrometer. GC (Agilent 7890B) equipped with an HP-5 column (30 m × 0.25 mm × 0.25 μm), and a flame ionization detector (FID) was used to determine the specific concentration or for the quantitative analysis of the various substances. The conversion of FF and the yield of the product (*e.g.*, GVL and IPL) were calculated from standard curves made with commercial samples using naphthalene as an internal standard. The concentration of the respective substance in the solution was calculated with the following equations:1
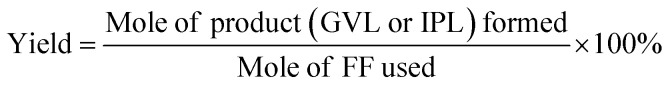
2
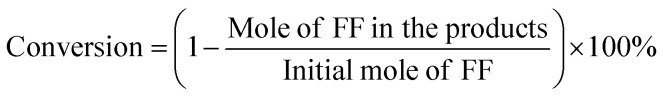


### 2.4 Catalyst characterization

FT-IR (Fourier transform infrared) spectra were recorded on a PerkinElmer 1710 spectrometer (KBr disc) in the wavenumber range of 400–4000 cm^−1^. XRD (X-ray diffraction) patterns were recorded on a Bruker D8 Advance system using Cu Kα radiation with 2*θ* = 5°–80°. XPS (X-ray photoelectron spectroscopy) analysis was conducted on a Physical Electronics Quantum 2000 Scanning ESCA Microprobe (Mono Al-Kα, *hν* = 1486.6 eV). The pass energy of the full-spectrum scan was 100 eV, and the pass energy of the narrow-spectrum scan was 60 eV. The XPS spectra were calibrated based on the surface contamination C 1s (284.8 eV). The residual Hf in the reaction liquid was tested by ICP-OES (inductively coupled plasma optical emission spectroscopy, Agilent 720). SEM (scanning electron microscopy) images were obtained using a ZEISS SIGMA300 system. The powder samples were bonded on conductive adhesive for the SEM measurements. HR-TEM (high-resolution transmission electron microscopy) images were obtained using an FEI TALOS F200C system, with a resolution of 0.16 nm. The TEM samples were dispersed in absolute ethanol after ultrasonic vibration and deposited on a carbon-coated copper grid.

## 3. Results and discussion

### 3.1 Effect of the reaction temperature and time

**Fig. 1:**
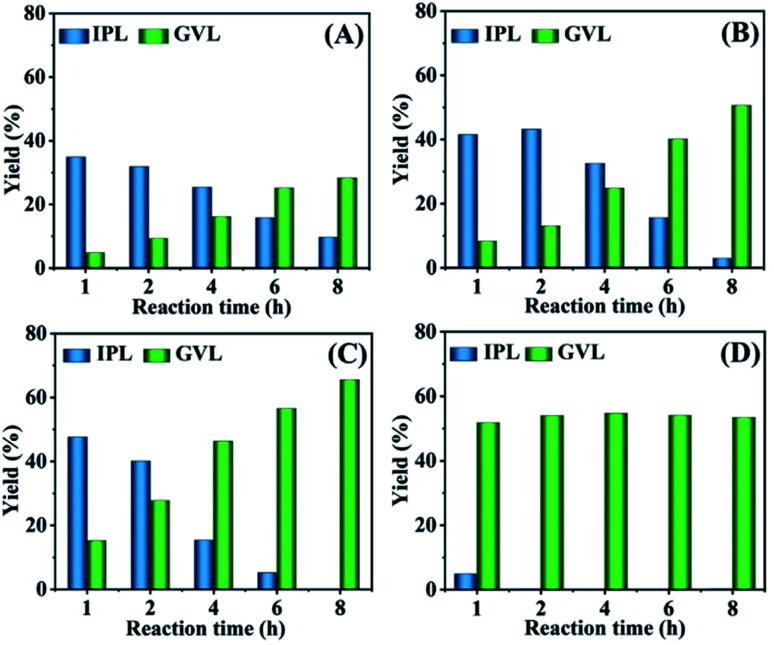
Effect of the reaction temperature and time on the conversion of FF to GVL (A: 413, B: 433, C: 453, D: 473 K). Reaction conditions: 1 mmol FF, 0.03 mol% HfCl_4_, 6 mL 2-propanol, 413–473 K for 1–8 h.

The effect of the reaction temperature on the cascade transformation of FF into GVL was investigated over HfCl_4_ at 413–473 K. FF could be almost completely transformed at the reaction temperature of 413–473 K in the reaction time range of 1 h to 8 h (Fig. S1†). As shown in Fig. 1, as the reaction temperature rose from 413–453 K (reaction for 1 h), the yield of GVL and IPL increased from 4.8% and 34.8% to 15.2% and 47.6%, respectively. This indicated that a higher temperature was favourable for the GVL synthesis from FF. When the reaction time was extended from 1 h to 8 h (at 453 K), the yield of GVL increased from 15.2% to 64.2%. Obviously, a longer reaction time and higher temperature were more conducive to the formation of GVL. In addition, it must be mentioned that IPL was the main by-product in all cases and could be gradually converted to GVL as the reaction time extended. This may be because the conversion of IPL to GVL required two processes: transfer hydrogenation and cyclization, and the isopropyl group is difficult to be removed in the cyclization reaction. Therefore, a higher temperature and longer reaction time were conducive to a better GVL yield. After heating to 473 K for 1 h, the yield of GVL reached 51.8% (Fig. 1D). However, the yield of GVL did not change significantly with the extension of the reaction time. It is speculated that a higher reaction temperature will promote the occurrence of side reactions, resulting in an increase in humin attached to the catalyst and a reduction in active sites that can be contacted. Therefore, continuing to extend the reaction time does not significantly increase the yield of GVL. In addition, an interesting phenomenon could also be observed, wherein the yield of GVL obtained at 473 K was lower than at 453 K. This is because the origin of the Lewis acid site (Hf^4+^) should be attributed to HfCl_4_, and HfO(OH)_2_·*x*H_2_O is gradually generated as the reaction progresses. A relatively high reaction temperature accelerates the reaction and also promotes the formation of HfO(OH)_2_·*x*H_2_O, because –OH in HfO(OH)_2_·*x*H_2_O may occupy the empty orbital of Hf^4+^, resulting in a decrease in the strength of the Lewis acid sites in the catalyst. Therefore, the yield of GVL decreased when the reaction temperature was too high.

### 3.2 Effect of the hydrogen source type

**Fig. 2:**
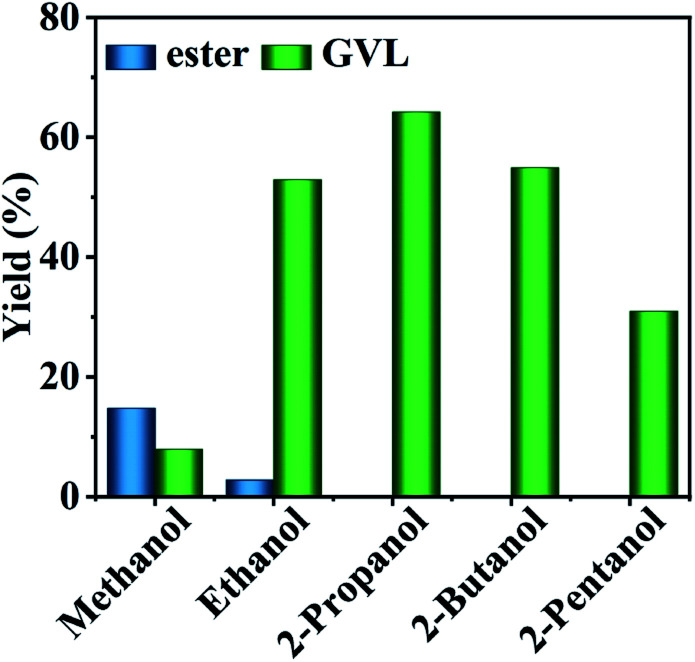
Conversion of FF to GVL with different alcohols. Reaction conditions: 1 mmol FF, 6 mL alcohol, 0.03 mol% HfCl_4_, 453 K, and 8 h.

The catalytic results of HfCl_4_ in the conversion of FF to GVL with different primary and secondary alcohols as hydrogen donors at 453 K for 8 h are shown in Fig. 2. When methanol was used as a hydrogen source, the yield of GVL was as low as 7.9%, with the IPL yield only 14.7%. On the contrary, ethanol showed a good hydrogen-donating ability, and the yield of GVL was as high as 52.9%, with the IPL yield as low as 2.8%. This may be due to the steric hindrance of methanol while ethanol is small, and the hydrogen supply capacity mainly depends on the reduction potential energy of alcohol. Table S1† provides data on the reduction potential energy and steric hindrance of various alcohols. It is obvious that ethanol has a much lower reduction potential energy than methanol (85.4 kJ mol^−1^*vs.* 130.1 kJ mol^−1^). Therefore, ethanol has a better hydrogen supply capacity than methanol.^8,32,42^ Moreover, the fact ethanol showed a better hydrogen supply capacity could also be ascribed to the fact that the transition state between the hydrogen donor and the catalyst was more stable with the extension of the carbon chain of the primary alcohol.^43^ For the secondary alcohol, 2-propanol revealed a higher GVL yield of up to 64.2%, demonstrating that it was a better hydrogen donor. This may be because the β-hydrogen of sec-alcohol is easier to be removed from the intermediate alcohol oxide than from the primary alcohol *via* the CTH reaction. With the extension of the alcohol carbon chain, the yield of GVL decreased significantly from 64.2% in 2-propanol, 54.9% in 2-butanol, to 31.0% in 2-pentanol. This may be because secondary alcohols have relatively larger steric hindrance, which makes it difficult to contact with the catalyst active sites to form a stable transition state, and thus is not conducive to the occurrence of the CTH reaction.^19^ Accordingly, the yield of GVL gradually decreased with the extension of the carbon chain length of the secondary alcohol. Hence, 2-propanol was selected as the optimal hydrogen donor.

### 3.3 Effect of the catalyst dosage

**Fig. 3:**
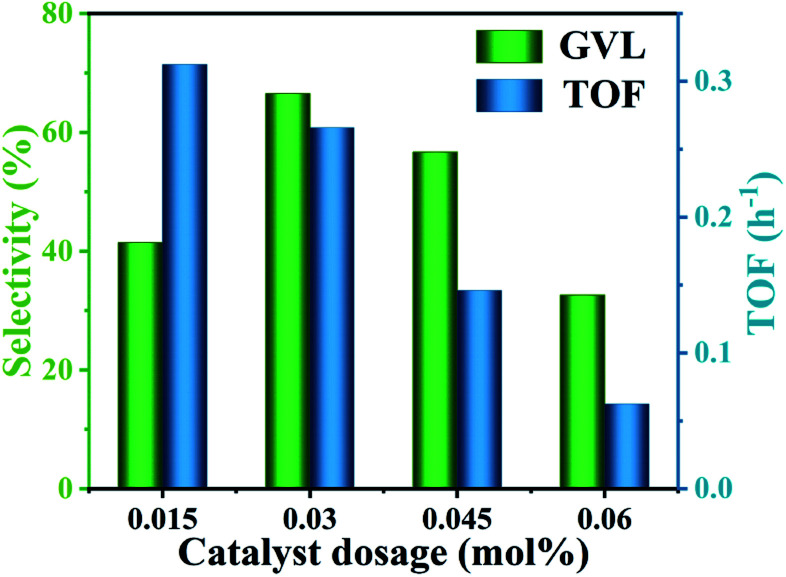
GVL yield and TOF value with different HfCl_4_ dosages. The TOF is defined as mol (formed GVL)/[mol (catalyst dosage) × h (time)]. Reaction conditions: 1 mmol FF, 6 mL 2-propanol, 453 K, and 8 h.

The excellent catalytic activity was mainly attributed to the content of Lewis acid–base sites and Brønsted acid sites available in the reaction system. Since the Lewis acid sites involved in the reaction in the reaction system were completely derived from Hf^4+^, the content of Lewis acid in the reaction system was consistent with the content of Lewis acid in HfCl_4_ (3.1 mmol g^−1^). Therefore, it is particularly important to select a proper amount of HfCl_4_ to obtain an enhanced GVL yield. As shown in Fig. 3, as the amount of HfCl_4_ catalyst increased from 0.015 mol% to 0.06 mol%, the yield of GVL increased from 36.5% to 64.2%. However, with further increasing the amount of HfCl_4_, the yield of GVL was significantly reduced. It is worth noting that the TOF value (from 0.31 to 0.06 h^−1^) showed a decreasing trend as the amount of HfCl_4_ increased. This is because when the amount of catalyst was too great, more catalyst was prone to agglomerate, further increasing the particle size of the catalyst and causing mass-transfer resistance. This may extend the residence time of the substrate and product in the catalyst, which will cause side reactions to form by-products (Fig. S2†).^22^ Therefore, TOF decreased as the amount of catalyst increased. This can explain why excessive HfCl_4_ cannot achieve higher GVL productivity. In general, when the catalyst dosage was 0.03 mol%, it could obtain a higher GVL yield (64.2%) although the TOF value (0.27 h^−1^) was slightly lower than 0.015 mol%. Therefore, 0.1 g was selected as the optimal catalyst dosage.

### 3.4 Response surface methodology analysis

To further investigate the effect of various factors for the one-pot conversion of FF to GVL, a three-factor and three-level central composition design of response surface analysis (RSA) was applied. The independent factors included the reaction temperature (A), reaction time (B), and catalyst dosage (C), and the GVL yield was designated as the related response factor. Table S2† shows the research range and corresponding level of each influencing factor based on the previous single-factor optimization. The Box–Behnken experimental design was performed by Design-Expert 8.0.6 software. Table S3† shows the reaction conditions of each group and the yield of GVL under these conditions. In addition, Fig. S3A† is the relative evaluation diagram of the experimental and predicted GVL yields, showing that the actual value was relatively close to the linear distribution. This demonstrates that there was almost no notable difference between experimental and predicted value.^12^ According to the above design results, the quadratic polynomial model of GVL yield was obtained, as shown in eqn (3):

3 GVL yield = +64.64 − 2.67*A* − 0.59*B* − 0.56*C* − 7.83*AB* − 4.58*AC* − 1.25*BC* − 7.71 *A*^2^ − 4.39*B*^2^ − 6.88*C*^2^

where *A*, *B*, and *C* represent the reaction temperature, reaction time, and catalyst dosage, respectively.

The standard analysis of variance (ANOVA) was conducted for this model to evaluate its sufficiency and goodness. It can be seen from the variance analysis results in Table 1 that the *P*-value below 0.05 suggested that this model was significant, and the variable significantly affected the response at the 95% confidence level. It is gratifying that on account of this analysis, the *P*-value of this model was less than 0.0001, suggesting that the model was highly significant. In addition, the lack of fit was not significant, exhibiting that the model fitted well with the actual process. In consequence, this equation could be effectively used to explore the influence of each factor in the reaction system on the yield of GVL. According to the *F* value of each influencing factor, the order of the factors important for the yield of GVL was *A* > *B* > *C*, that is, the reaction temperature had the largest influence relative to the other factors. This may be due to the conversion of IPL to GVL requiring a higher temperature during the reaction. Interestingly, *AB* > *AC* > *BC*, indicating that the interaction term of the reaction temperature and time had the greatest effect on the generation of GVL, while the interaction term of the catalyst dosage and reaction time had little effect on the yield of GVL.

**Table 1:** Analysis of variance (ANOVA) for the response surface quadratic model

Source	Sum of squares	d*f*	Mean square	*F* value	*P*-value prob. > *F*	Significant
Model	1006.32	9	111.81	42.43	<0.0001	Significance
*A*: reaction temperature	57.24	1	57.24	21.73	0.0023	
*B*: reaction time	2.76	1	2.76	1.05	0.3401	
*C*: catalyst dosage	2.53	1	2.53	0.96	0.3597	
*AB*	244.92	1	244.92	92.95	<0.0001	
*AC*	83.72	1	83.72	31.77	0.0008	
*BC*	6.25	1	6.25	2.37	0.1674	
*A* ^2^	257.48	1	257.48	97.72	<0.0001	
*B* ^2^	85.07	1	85.07	32.29	0.0007	
*C* ^2^	206.02	1	206.02	78.19	<0.0001	
Residual	18.44	7	2.63			
Lack of fit	17.13	3	5.71	17.41	0.0093	Not significant
Pure error	1.31	4	0.33			
Cor. total	1024.76	16				

A perturbation plot can connect all the factors in the response surface. While keeping other factors constant, the response surface is plotted by changing a factor. The slope or curvature indicates the sensitivity of the response value to this factor. The perturbation plot of this study is displayed in Fig. S3B.† It can be noticed that the influences of the three factors on the response value were all positive at a low level, but changed into negative impacts after increasing them up to a certain extent. This variation process was similar to the results of the previous single-factor optimization, which further supported the previous analysis of the impact of a single factor. Among these, the curvature corresponding to the amount of catalyst was the smallest, indicating that this factor had less influence compared with the other factors.

**Fig. 4:**
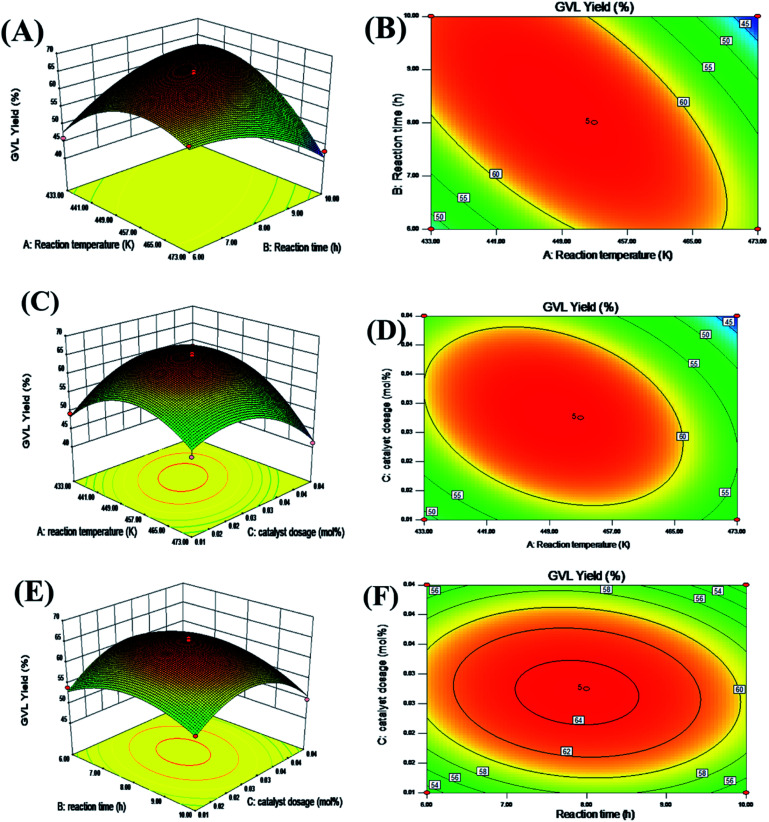
Reaction temperature, reaction time, catalyst dosage interaction 3D response surface (A, C, E), and contour map (B, D, F).

Fig. 4 presents a 3D and contour plot of the interaction of each independent factor on the GVL yield obtained in the experiment. The influence of the interaction effect of the different factors on the target value can be intuitively perceived from the inclination of the corresponding 3D diagram and the trend of the contour. The flatter the 3D graph, the smaller the effect of the corresponding factors on the response value. The ellipticity of the contour represents whether the interaction is significant. The closer the contour is to the circle, the less significant the interaction is ref. 43–46.

It can be seen from the contour plot that the interaction between the reaction time and temperature was the most obvious, and the slope of the corresponding 3D plot was also larger, which further showed that these two variables had a greater impact on the yield of GVL. In addition, whether it is a contour diagram or a 3D diagram, it can be seen that the yield of GVL was lower when the level of each reaction parameter was lower. This may be because when the amount of catalyst was small, the acid–base sites contained in the reaction system were not enough to catalyze the complete conversion of FF to GVL in a shorter reaction time and lower temperature. With the increase in the level of each reaction parameter, the yield of GVL gradually increased. Nevertheless, when the level of each reaction parameter increased to a certain extent, the yield of GVL began to decrease. This may be because as the catalyst amount increases, excessive HCl in the reaction system will cause a series of side reactions to occur. In addition, too high a reaction temperature will also cause changes in the active sites of the catalyst, so extending the reaction time cannot obtain a higher GVL yield.

The optimal reaction conditions and the highest GVL yield were obtained by RSM. That is, when the amount of catalyst was 0.03 mol%, the GVL yield reached the highest at 64.7% at 447.8 K for 8.34 h. After that, we verified the optimized conditions. When the dosage of FF was 1 mmol and 0.03 mol% catalyst was used, the yield of GVL obtained by reacting at 448 K for 8.3 h was 64.5%. Compared with the predicted value, there was only a 0.3% error, so the established model could reflect the actual situation well. Notably, this result required a shorter reaction time than in many other reports (Table 2), which can greatly improve the production efficiency. Combined with the above analysis, it is considered that this is because the reaction system contained a moderate intensity of Lewis acid–base sites, which made the CTH reaction in the process of FF-to-GVL conversion fully carried out. At the same time, the Brønsted acid (HCl) produced by the hydrolysis of HfCl_4_ could effectively catalyze the rapid ring-opening reaction of the furan ring. Therefore, an excellent GVL yield could be obtained after only 8.3 h of reaction. More importantly, the commercial catalyst could obtain GVL yields similar to those of lab synthesis catalysts, and did not require complicated and lengthy preparation processes. From a price point of view, the lab preparation of catalysts often requires commercial catalysts as raw materials, so it is more economical than lab synthesis catalysts. Overall, this work provides a feasible reference in principle for industrial scale.

**Table 2:** Catalytic results of FF-to-GVL conversion in previous reports and in this work

Entry	Catalyst	H-donor	Temp. (K)	Time (h)	Conv. (%)	Yield (%)	Ref.
1	HPW/Zr-beta	2-Propanol	453	24	100	68	26
2	Zr/T/zeolite	2-Propanol	443	10	100	85	27
3	Zr-CN/H-β	2-Propanol	433	18	100	76.5	28
4	ZrO_2_-[Al]MFI-NS-30	2-Propanol	443	36	100	82.8	29
5	DUT-67(Hf)	2-Propanol	453	24	100	87.1	30
6	HZ-ZrP1-16	2-Propanol	458	18	100	64.2	31
7	ZPS-1.0	2-Propanol	423	18	100	80.4	32
8	HfCl_4_	2-Propanol	448	8.3	100	64.5	This work

### 3.5 Kinetics study

**Fig. 5:**
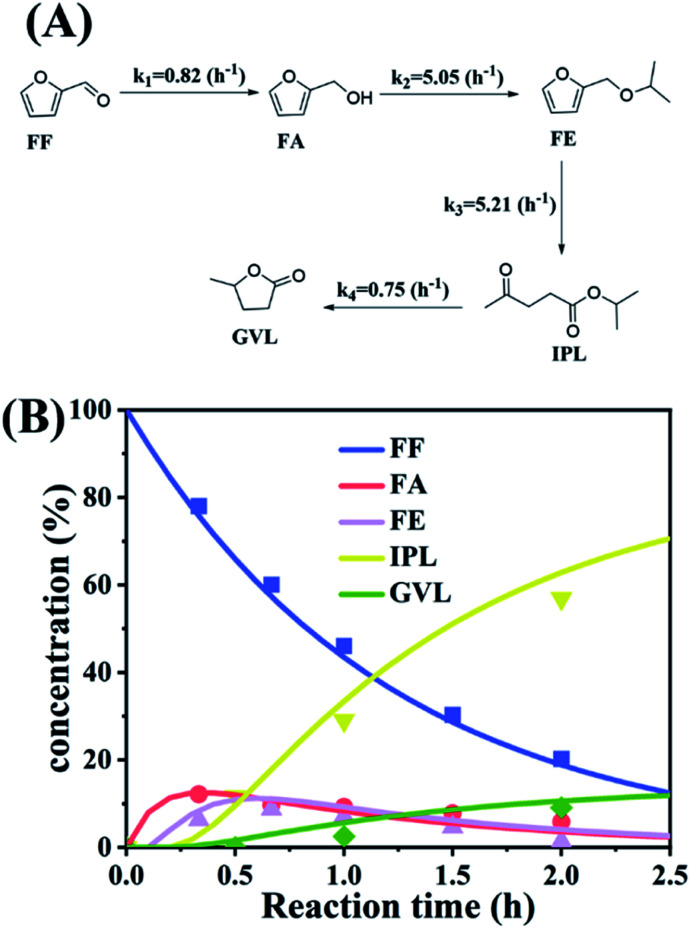
(A) Kinetic model for the conversion of FF to GVL, and (B) the concentration of each substance in the kinetic model varying with the reaction time and the corresponding kinetic fitting curve at 403 K.

To further understand the conversion of FF to GVL catalyzed by HfCl_4_ in 2-propanol, we studied the kinetic process of FF conversion to FA, FE, IPL, and GVL based on the one-pot method, and established a simplified kinetic model based on the whole process (Fig. 5A). The kinetic experiment was carried out at 403 K in order to study the reaction rate of each process involved in the conversion of FF to GVL. In the entire reaction system, the amount of 2-propanol in the reaction system was excessive relative to the substrate (78.5 mmol *vs.* 1 mmol), so it could be assumed that the concentration of 2-propanol was constant in the reaction process. Therefore, a pseudo first-order kinetic model was used to calculate the reaction rate constant of each reaction step. Thus, the relationship between the substrate conversion rate and the reaction time can be expressed as the following reaction rate equations:4
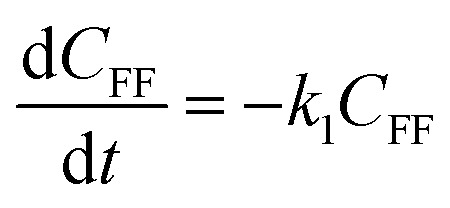
5
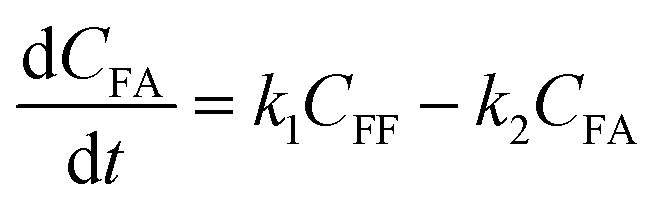
6
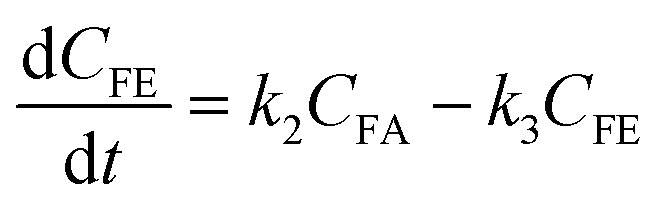
7
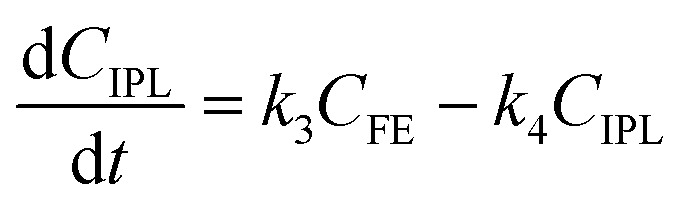
8
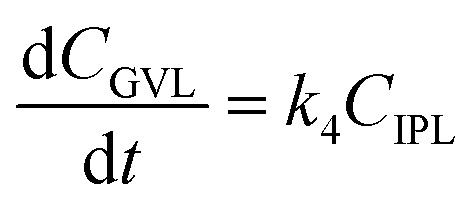
where *k*_1_, *k*_2_, *k*_3_, and *k*_4_ are the reaction rate constants of each step at a certain reaction temperature, (*t*) is the reaction time (h), and *C*_FF_, *C*_FA_, *C*_FE_, *C*_IPL_, and *C*_GVL_ represent the concentration of FF, FA, FE, IPL, and GVL, respectively.

The experimental results were fitted by the above equations, and the reaction rate constants of each step were obtained (Fig. 5B). It was observed that the reaction rate constant of IPL being converted to GVL was the smallest, indicated that this step was the rate-determining step in this reaction process of FF-to-GVL conversion. In addition, *k*_2_ and *k*_3_ were much larger than *k*_1_ and *k*_4_, indicating that FA and FE were rapidly converted during the reaction, which can explain why only the intermediate product of IPL was detected after the reaction was completed and FA and FE were not detected. The main reason for this result may be that the HCl generated by the hydrolysis of HfCl_4_ is a strong Brønsted acid, which can effectively promote the etherification reaction between FA and 2-propanol and the ring-opening process of FE to generate IPL. The lower reaction rate constant of IPL may be because in the reaction progresses, HfCl_4_ is gradually converted to HfO(OH)_2_·*x*H_2_O, which has a weaker Lewis acid strength, resulting in a slower progress of the CTH reaction.

### 3.6 Reaction mechanism

**Scheme 2:**
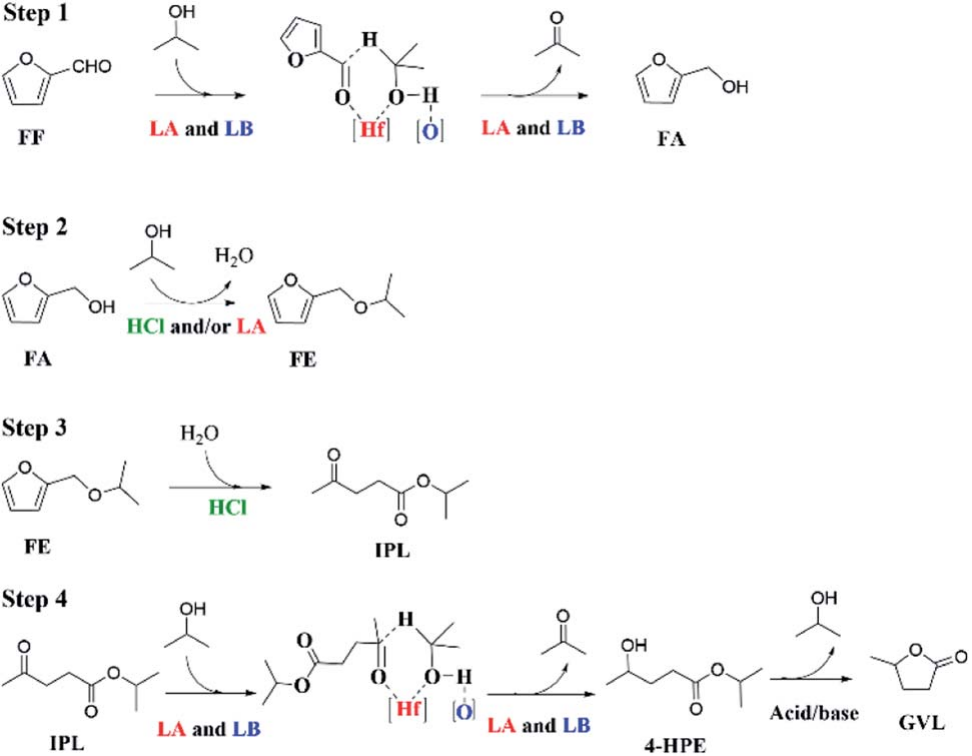
Possible reaction pathways for the cascade conversion of FF to GVL in one pot (LA = Lewis acid, LB = Lewis base).

According to the above experimental and corresponding results discussions, a feasible reaction path for the transformation of FF into GVL through a one-pot cascade reaction process is proposed (Scheme 2). In the CTH process, Lewis acid and base sites play a synergistic role.^37^ HfCl_4_ provides Lewis acid sites (Hf^4+^), while HfO(OH)_2_·*x*H_2_O and HCl are gradually *in situ* generated by HfCl_4_ hydrolysis in 2-propanol due to the presence of residual water (content: *ca.* 0.5%) to provide Lewis base sites (O^2−^) and Brønsted acid sites (HCl), respectively, as illustrated by FT-IR (Fig. S4†). At the beginning of the reaction process, the carbonyl group of FF is adsorbed on the Lewis acid site (Hf^4+^), and 2-propanol with the oxygen and hydrogen atom of the hydroxyl group are respectively adsorbed onto the Lewis acidic Hf^4+^ and the Lewis basic oxo-ion site.^40^ Then, a six-membered ring transition state is formed to complete the transfer hydrogenation process. FF is converted to FA, and at the same time the 2-propanol is transformed to acetone. After that, FA reacts with 2-propanol catalyzed by the Lewis acid sites to form isopropyl furfuryl ether (FE) by an etherification reaction and producing the same amount of water (1 mmol). A part of the formed water will be consumed by HfCl_4_ hydrolysis to *in situ* generate more HfO(OH)_2_·*x*H_2_O in moderate basicity and HCl with strong Brønsted acidity. Due to the HCl generated in the reaction system being sufficient, this process proceeds quickly, which is consistent with the large reaction rate constant of FA. Since the subsequent ring-opening reaction of FE also occurs under the action of HCl, this process is also easy to occur. Finally, IPL transformation into GVL requires two steps: transfer hydrogenation and cyclization reactions. The conversion of IPL to isopropyl 4-hydroxyvalerate (4-HPE) *via* transfer hydrogenation is similar to that of FF to FA, which is also catalyzed by the Lewis acid–base site (Hf^4+^–O^2−^). Finally, the cyclization of 1 mmol 4-HPE to produce equivalent GVL is conducted in the presence of acid sites. However, the process of generating GVL from IPL is the most difficult to carry out, showing it is the rate-determining step of the transfer hydrogenation and cyclization.

### 3.7 Catalyst reusability

The filtration test was carried out before the catalyst reusability test. As displayed in Fig. S5,† it can be obviously noticed that after the catalyst was filtered, the yield of GVL almost did not change with prolonging the reaction time, indicating that the formation of GVL was performed in a heterogeneous manner. In addition, the ICP results showed that the residual Hf in the mixture could be ignored (0.0002 mg L^−1^), which strongly demonstrated that the leaching of Hf in the reaction process could be ignored, so the catalyst has the potential to be recycled.

**Fig. 6:**
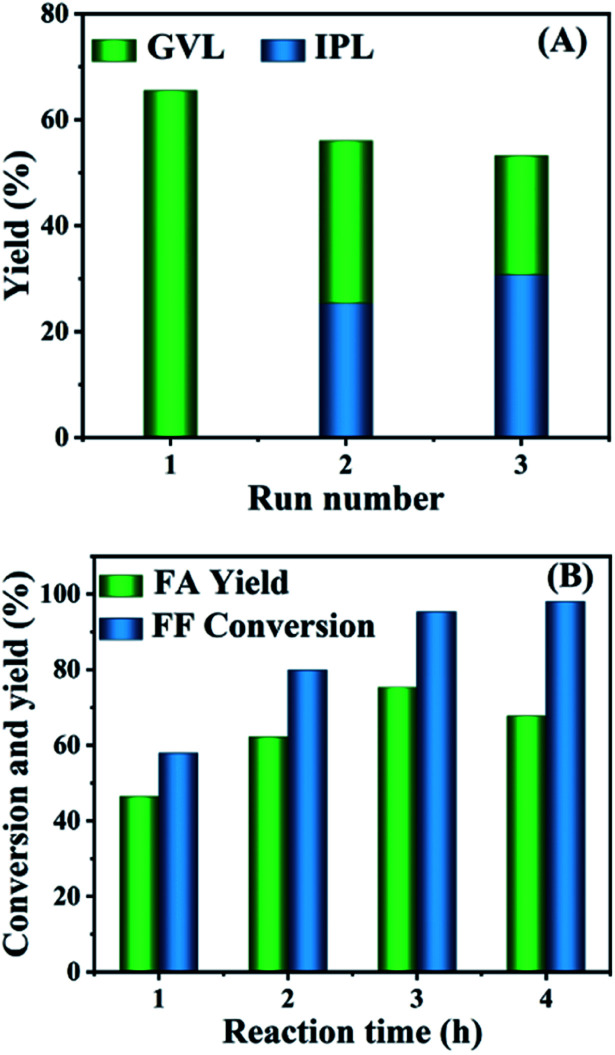
Catalyst reusability in the conversion of FF to GVL (A); and the HfO_2_-(c) performance in the transformation of FF into FA (B); reaction conditions: 1 mmol FF, 0.03 mol% HfO_2_-(c), 6 mL 2-propanol, 433 K.

After the cascade conversion of FF to GVL catalyzed by HfCl_4_, the catalyst was collected by centrifugal separation, washed twice with ethyl alcohol, and dried at 353 K in an oven. Since the main component of the recovered catalyst was HfO(OH)_2_·*x*H_2_O, its Lewis acidity was weaker than HfCl_4_ and it could not effectively catalyze the synthesis of GVL from FF. Therefore, it was necessary to regenerate HfCl_4_ to increase the Lewis acidity of the catalyst to promote the reaction. The activated catalyst was used for the selective conversion of FF to GVL, and the results are shown in Fig. 6A. FF was completely converted in each cycle, and the yield of GVL was significantly reduced, while the carbon balance of the reaction system was only slightly reduced. The decrease in GVL yield was due to the fact that although the Lewis acidity of the recovered catalyst increased after activation, the humin attached to the catalyst could not be completely removed, resulting in a decrease in the active sites that could be contacted. Therefore, compared with fresh catalyst, the GVL yield obtained by the recovered catalyst was reduced. However, since the content of the main active sites (Hf^4+^) did not decrease significantly, the carbon balance did not change remarkably compared with the fresh catalyst, but with more IPL.

Because the hydrolysis of HfCl_4_ to HfO(OH)_2_·*x*H_2_O occurred simultaneously with the formation of humin, it could be considered that the humin generated *in situ* during the reaction process could be used as a templated surfactant for the generation of HfO(OH)_2_·*x*H_2_O.^43^ Considering that HfO(OH)_2_·*x*H_2_O can be calcined to obtain HfO_2_, HfO_2_ containing Lewis acid–base sites can successfully catalyze the CTH reaction of aldehydes/ketones. Therefore, calcining the collected solid at high temperature can be envisaged to remove the humin in the solid, and the obtained catalyst may then catalyze the transfer hydrogenation of aldehydes/ketones. Therefore, the collected solid was calcined in a tubular muffle furnace at 723 K for 6 h to obtain the target catalyst. For the convenience of the research, we named the catalyst before calcination as HfO_2_-(b) and the catalyst after calcination as HfO_2_-(c). Under other identical reaction conditions, HfO_2_-(c) was used to catalyze the conversion of FF to FA. As shown in Fig. 6B, at 433 K, as the reaction time increased, the conversion rate of FF and the yield of FA increased. When the reaction time was extended to 3 h, the productivity of FA achieved 75.3%. With further extending the reaction time, the yield of FA began to decrease, leading to the formation of ether.

### 3.8 Catalyst characterization

**Fig. 7:**
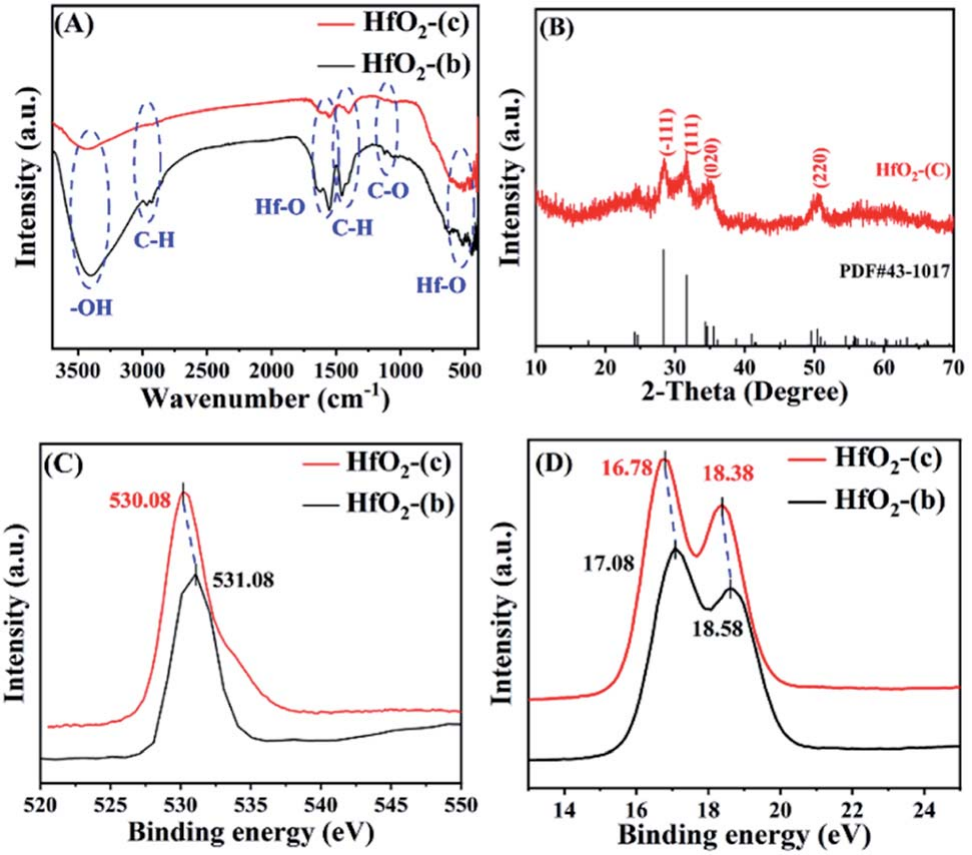
FT-IR spectra (A) of HfO_2_-(b) and HfO_2_-(c), XRD patterns (B) of HfO_2_-(c), and XPS patterns of O 1s (C) and Hf 4f (D).

The structure of the used catalysts before and after calcination was studied. As shown in Fig. 7A, the absorption peak near 3400 cm^−1^ is the stretching vibration of –OH, which may be attributed to the incomplete removal of –OH part of Hf in HfO(OH)_2_·*x*H_2_O.^47^ The absorption peak at near 2975 cm^−1^ can be attributed to C–H stretching vibration, while the absorption peak at 1450 cm^−1^ is C–H bending vibration, and the absorption peak at 1056 cm^−1^ may be C–O stretching vibration.^48^ These absorption peaks in HfO_2_-(c) were significantly reduced, indicating that most of the humin in HfO_2_-(b) was removed after the calcination. The absorption peaks at 1600 cm^−1^ and 530 cm^−1^ are the bending vibration and stretching vibration of the Hf–O bond, respectively, indicating that HfO_2_ was indeed generated after the catalyst was calcined.

**Fig. 8:**
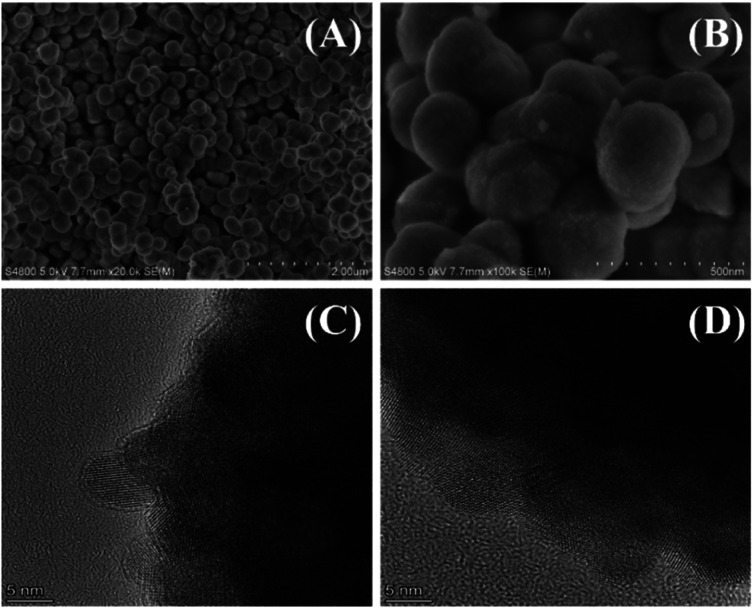
SEM (A and B) and HR-TEM (C and D) images of HfO_2_-(c).

The XRD spectrum of the catalyst after calcination (Fig. 7B) showed an obvious absorption peak, which has high agreement with the standard card PDF # 43-1017 of HfO_2_ (ref. 48), indicating that the collected catalyst had good crystallinity after calcination. Then, the morphology of HfO_2_-(c) was studied by SEM and HR-TEM images. The SEM images (Fig. 8A and B) showed that HfO_2_-(c) comprised spherical particles with good dispersion and a uniform particle size. This is because of the role of humin as a template and as the self-assembly of the catalyst occurred before calcination, whereby the catalyst will exhibit a spherical shape after calcination to remove the humin. The HR-TEM images (Fig. 8C and D) clearly show that HfO_2_-(c) had different crystal orientations, which is consistent with the crystal diffraction peaks observed in the XRD images of HfO_2_-(c) materials.

It has been reported that the intensity of the Lewis acid–base sites in a catalyst has a great impact on the MPV reduction reaction.^47,49,50^ Therefore, XPS diagrams of the collected catalyst and the HfO_2_-(c) were analyzed to explore the changes in the intensity of the acid–base sites in the catalyst. Fig. S6† presents the XPS survey spectra, and the XPS spectra of C 1s are shown in Fig. S7,† with the binding energy of O 1s and Hf 4f in HfO_2_-(c) shown in Fig. 7C and D, respectively. No significant change was observed in the binding energy for C 1s of the catalysts before and after being calcination. Obviously, the binding energy of O 1s decreased after the catalyst was calcined, which may be caused by the electron cloud density around O atoms in Hf–OH (HfO_2_-(b)) being lower than that in Hf–O (HfO_2_-(c)). Therefore, the negative charge density of O atoms in HfO_2_-(c) was higher, thus showing a stronger Lewis basicity. Similarly, it can be seen that the Hf 4f binding energies of HfO_2_-(c) at 16.78 eV and 18.38 eV were slightly lower than the collected catalyst at 17.08 eV and 18.58 eV, respectively, indicating that the density of positive charges around Hf atoms in HfO_2_-(c) was relatively small, which resulted in the relatively low Lewis acidity.^48^ In the MPV reduction reaction process, the Lewis acid site is mainly used to complete the transfer hydrogenation process, and the Lewis base site mainly plays the role in activating the hydroxyl group. Therefore, it was demonstrated that HfO_2_-(c) can effectively catalyze the production of FA from FF.

## 4. Conclusions

In summary, HfCl_4_ was developed as an efficient bifunctional catalyst for the selective upgrading of FF to GVL with a good performance in a one-pot process. When 2-propanol was used as a hydrogen donor and solvent, the yield of GVL reached 64.2% at 453 K for 8 h. HfCl_4_ exhibited excellent catalytic activity due to the Lewis acidity of HfCl_4_ and moderate basicity of HfO(OH)_2_·*x*H_2_O, as well as the strong Brønsted acid generated by the *in situ* hydrolysis. RSM was used to optimize the reaction system, and the reaction temperature was found to have the greatest influence on the yield of GVL. This may be because the processes of transfer hydrogenation and IPL-to-GVL conversion are easier to perform at a high temperature. Apart from the hydrogen transfer process, kinetic experiments also showed that the process of IPL-to-GVL conversion was the rate-determining step for the whole course of this reaction because the removal of the isopropyl group was difficult in the cyclization process. In addition, the catalyst after the reaction was activated by HCl and could be reused with good activity. Interestingly, HfO_2_-(c) obtained by calcination of the recovered catalyst in the air could effectively catalyze the conversion of FF to FA through cascade conversion processes. The *in situ* bifunctional catalyst is promising for the catalytic hydrogen transfer of biomass-derived aldehydes/ketones in combination with other reaction steps in a single one-pot process.

## Conflicts of interest

There are no conflicts to declare.

## Supplementary Material

RA-011-D1RA05637A-s001
