# Supplementary material for: One-step upgrading of bio-based furfural to γ-valerolactone via HfCl4-mediated bifunctional catalysis
Source: RSC Adv. 2021 Nov 2;11(56):35415–24. doi: 10.1039/d1ra05637a (PMC9043276; doi:10.1039/d1ra05637a)
Supplement: RA-011-D1RA05637A-s001 [file RA-011-D1RA05637A-s001.pdf]

Electronic Supplementary Material (ESI) for RSC Advances.
This journal is © The Royal Society of Chemistry 2021

Electronic supplementary information

One-step upgrading of bio-based furfural to γ-valerolactone via $HfCl_4$-mediated bifunctional catalysis

Mingrui Li, Yixuan Liu, Xialing Lin, Jinyu Tan, Song Yang*, Hu Li*

State Key Laboratory Breeding Base of Green Pesticide & Agricultural Bioengineering, Key Laboratory of Green Pesticide & Agricultural Bioengineering, Ministry of Education, State-Local Joint Laboratory for Comprehensive Utilization of Biomass, Center for Research & Development of Fine Chemicals, Guizhou University, Guiyang, Guizhou 550025, China

* Corresponding authors.

E-mails: hli13@gzu.edu.cn (HL); jhzx.msm@gmail.com (SY)

Table S1 The reduction potential ($\Delta_f H^o$) and steric hindrance of various reducing alcohols (hydrogen donors).

Entry	Reducing alcohols	$\Delta_f H^o$ (kJ/mol)	Steric hindrance (kJ/mol)
1	methanol	130.1 [a]	--
2	ethanol	85.4 [a]	11.8 [c]
3	2-propanol	70.0 [b]	17.2 [c]
4	2-butanol	69.3 [b]	22.2 [c]

[a] Data from van der Waal et al. (S1). [b] Data from van der Waal *et al*. (S2). [c] Data from van der Waal *et al*. (S3).

Table S2 Factors and levels in the response surface test.

Factor	Coded		
	-1	0	1
A: reaction temperature (K)	433	453	473
B: reaction time (h)	6	8	10
C: catalyst dosage (mol%)	0.015	0.03	0.045

Table S3 Center composition design matrix together with the experimental response values.

Run	A/K	B/h	C/mol%	GVL Yield/%
1	453	10	0.015	54.4
2	453	8	0.03	65.5
3	433	10	0.03	61.4
4	453	10	0.045	49.8
5	473	8	0.015	50.5
6	453	8	0.03	64.2
7	433	8	0.015	49.3
8	453	8	0.03	64.1
9	473	8	0.045	41.2
10	453	6	0.045	54.4
11	433	6	0.03	46
12	433	8	0.045	58.3
13	453	8	0.03	64.9
14	453	8	0.03	64.5
15	453	6	0.015	54
16	473	6	0.03	58.9
17	473	10	0.03	43

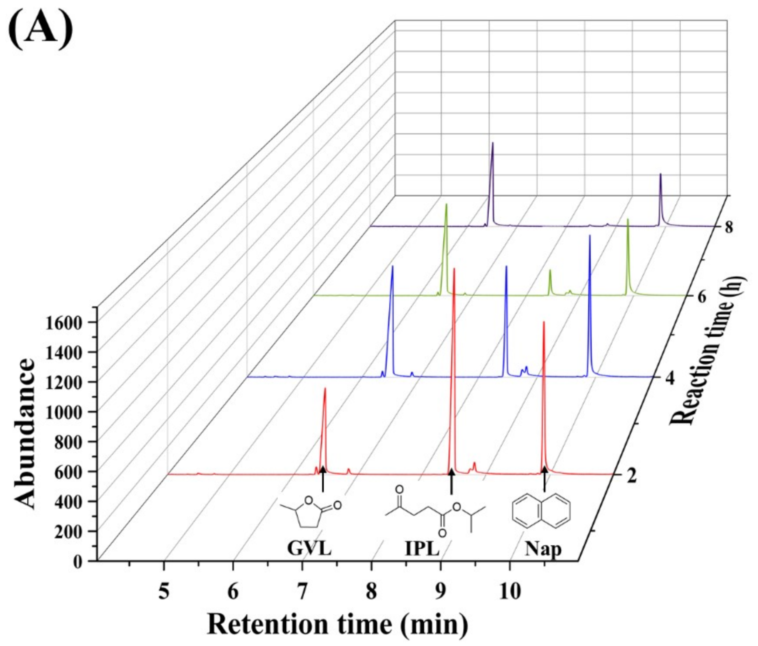

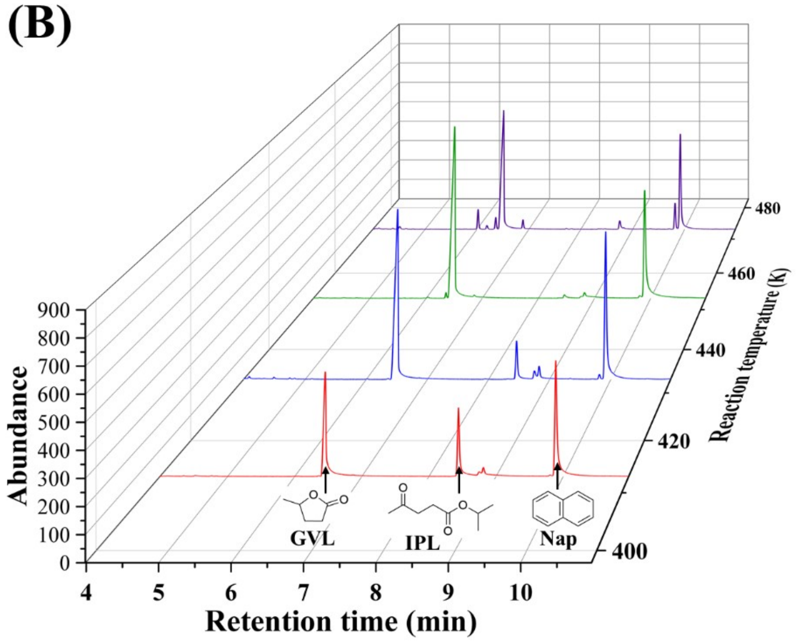

Fig. S1 GC spectra of reaction mixtures (FF-to-GVL conversion) after different reaction times at 453 K (A), and GC spectra of the reaction mixtures (FF-to-GVL conversion) after 8 h at different temperatures (B).

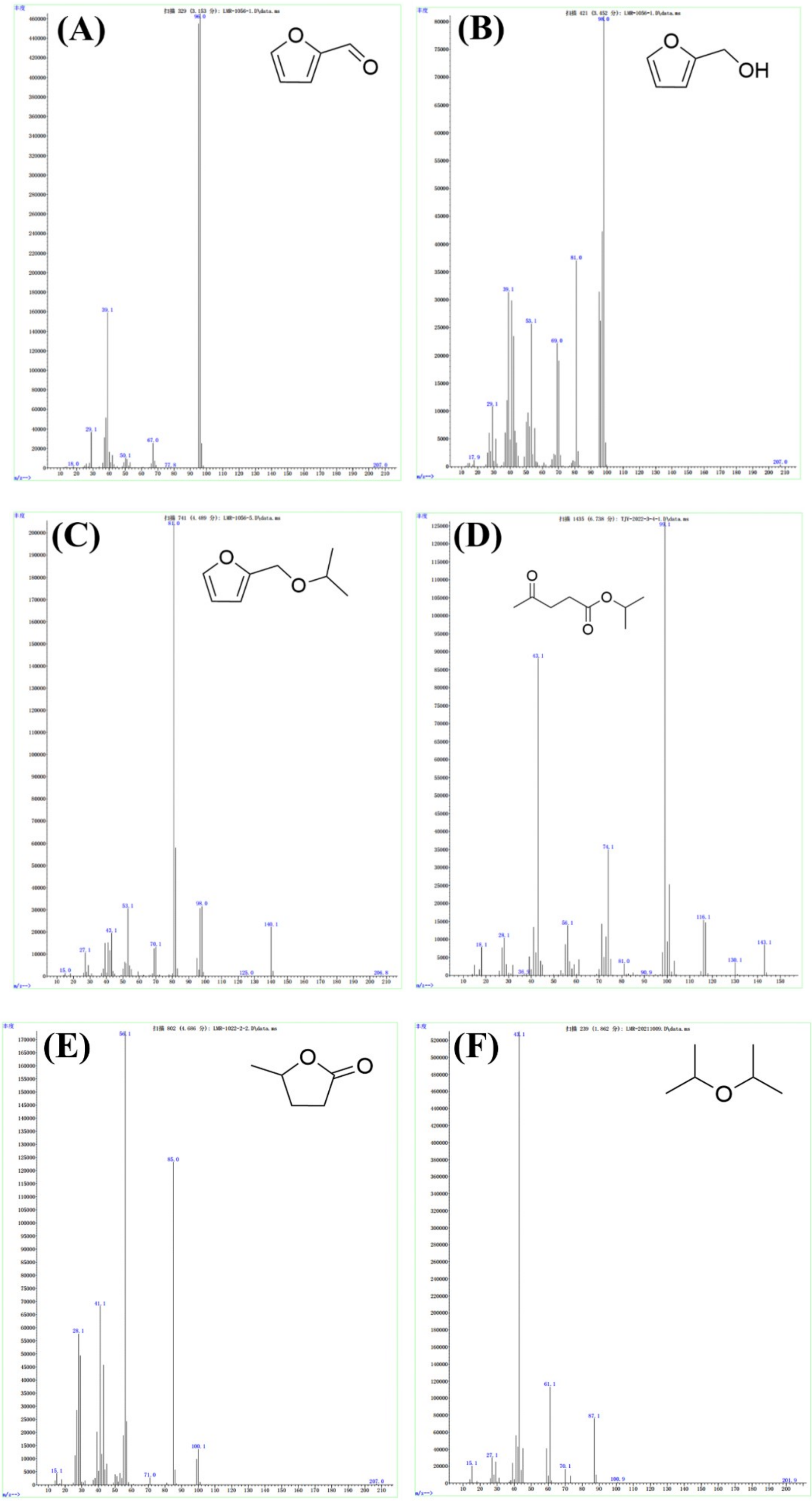

Fig. S2 GC-MS spectra of FF (A), FA(B), FE (C), IPL (D), GVL (E) and isopropyl ether (F).

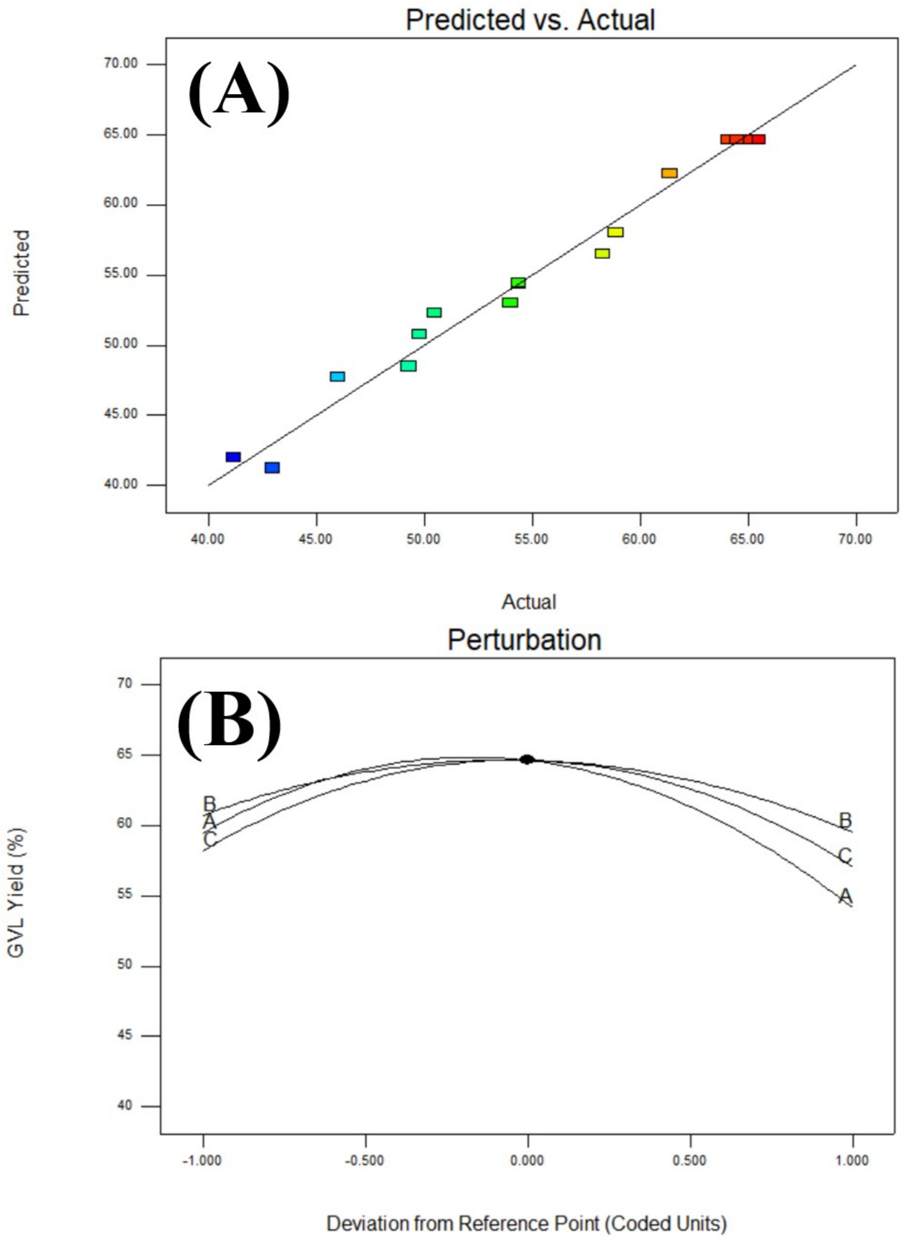

Fig. S3 Parity plot between actual and model predicted of GVL yield (A), and perturbation slope of all factors (B)

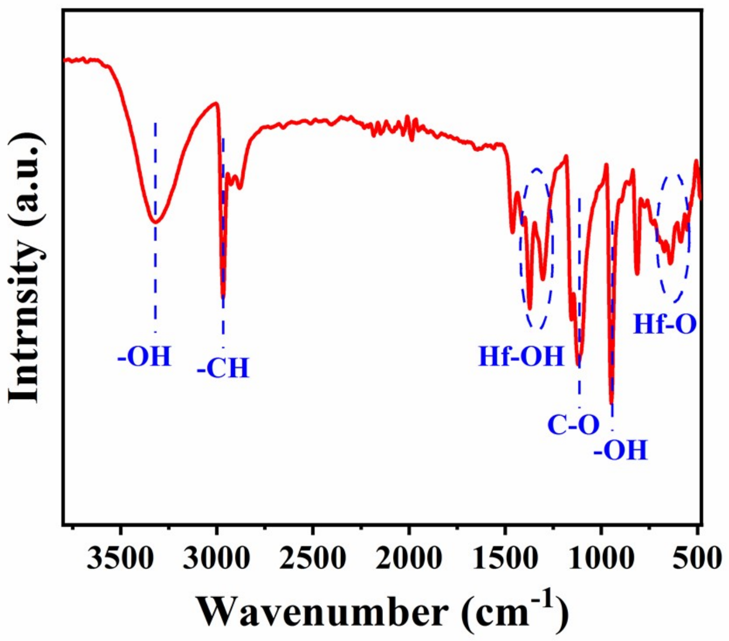

Fig. S4 FT-IR spectrum of the catalyst $HfCl_4$ after thermal treatment with 2-propanol (Reaction conditions: 0.3 mmol $HfCl_4$, 6 mL 2-propanol, at 403 K for 20 min).

Obvious Hf-OH and Hf-O absorption peaks are observed in the FT-IR spectrum (Fig. S4), indicating that the hydrolysis of $HfCl_4$ takes place in 2-propanol to form $HfO(OH)_2 \cdot xH_2O$, mainly with residual water in the commercial 2-propanol during the thermal treatment.

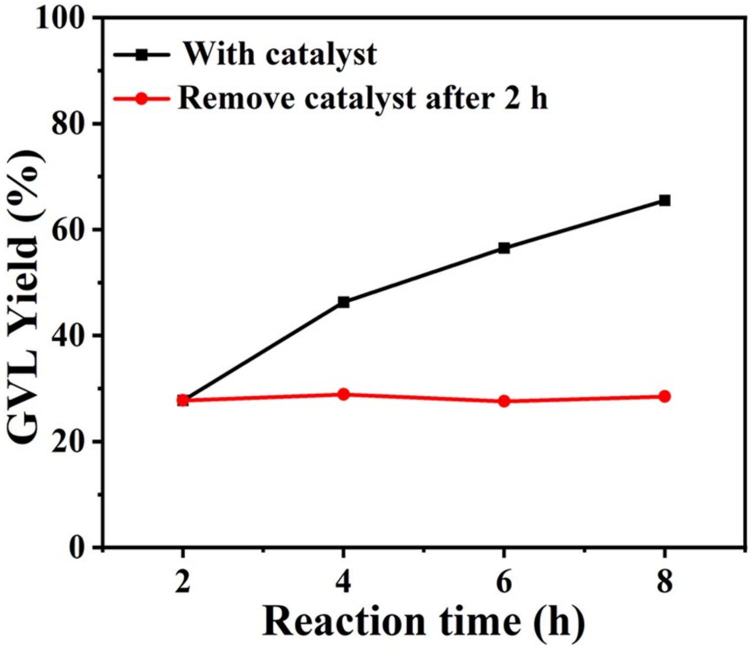

Fig. S5 GVL yield profiles of the reaction mixture with or without the catalyst (removed after 2 h) at 453 K.

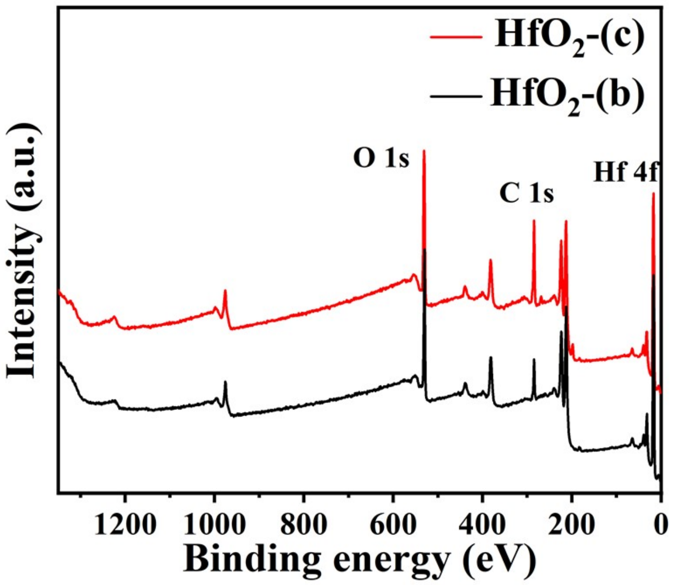

Fig. S6 The XPS survey spectra of the HfO_2-(b) and HfO_2-(c).

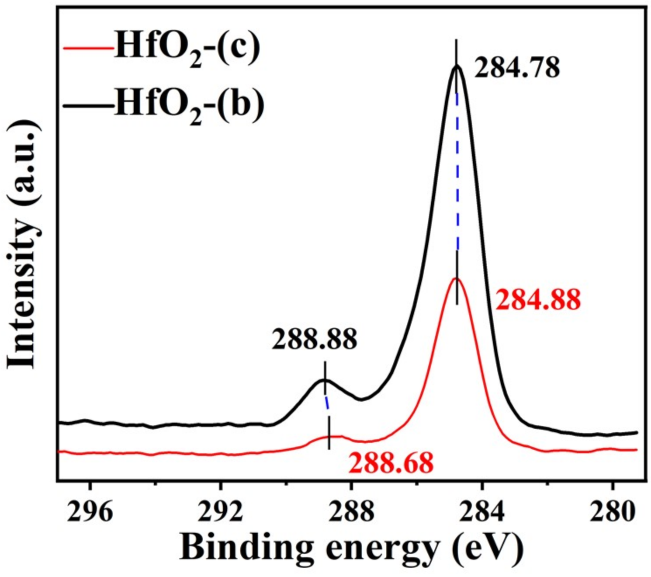

Fig. S7 The XPS spectra for C1s of the HfO_2-(b) and HfO_2-(c).

References:

[S1] X. Tang, H. Chen, L. Hu, W. Hao, Y. Sun, X. Zeng, L. Lin and S. Liu, *Appl. Catal. B: Environ.*, 2014, **147**, 827-834.

[S2] J.C. van der Waal, P.J. Kunkeler, K. Tan, H. van Bekkum, *J. Catal.*, 1998, **173**, 74-83.

[S3] W. Li, M. Li, H. Liu, W. Jia, X. Yu, S. Wang, X. Zeng, Y. Sun, J. Wei, X. Tang and L. Lin, *Mol. Catal.*, 2021, **506**, 111538.
